# Development of a Telemetry and Yield-Mapping System of Olive Harvester

**DOI:** 10.3390/s150204001

**Published:** 2015-02-10

**Authors:** Francisco J. Castillo-Ruiz, Manuel Pérez-Ruiz, Gregorio L. Blanco-Roldán, Jesús A. Gil-Ribes, Juan Agüera

**Affiliations:** 1 Dpto. de Ingeniería Rural, Universidad de Córdoba, Área de Mecanización y Tecnología Rural, Córdoba 14005, Spain; E-Mails: fcocastillo.agro@gmail.com (F.J.C.-R.); ir3blrog@uco.es (G.L.B.-R.); gilribes@uco.es (J.A.G.-R.); jaguera@uco.es (J.A.); 2 Aerospace Engineering and Fluid Mechanics Department, University of Seville, Ctra. Sevilla-Utrera km 1, 41013 Seville, Spain

**Keywords:** remote data acquisition, precision agriculture, effective field capacity, field efficiency

## Abstract

Sensors, communication systems and geo-reference units are required to achieve an optimized management of agricultural inputs with respect to the economic and environmental aspects of olive groves. In this study, three commercial olive harvesters were tracked during two harvesting seasons in Spain and Chile using remote and autonomous equipment that was developed to determine their time efficiency and effective based on canopy shaking for fruit detachment. These harvesters work in intensive/high-density (HD) and super-high-density (SHD) olive orchards. A GNSS (Global Navigation Satellite System) and GSM (Global System for Mobile Communications) device was installed to track these harvesters. The GNSS receiver did not affect the driver’s work schedule. Time elements methodology was adapted to the remote data acquisition system. The effective field capacity and field efficiency were investigated. In addition, the field shape, row length, angle between headland alley and row, and row alley width were measured to determinate the optimum orchard design parameters value. The SHD olive harvester showed significant lower effective field capacity values when alley width was less than 4 m. In addition, a yield monitor was developed and installed on a traditional olive harvester to obtain a yield map from the harvested area. The hedge straddle harvester stood out for its highly effective field capacity; nevertheless, a higher field efficiency was provided by a non-integral lateral canopy shaker. All of the measured orchard parameters have influenced machinery yields, whether effective field capacity or field efficiency. A saving of 40% in effective field capacity was achieved with a reduction from 4 m or higher to 3.5 m in alley width for SHD olive harvester. A yield map was plotted using data that were acquired by a yield monitor, reflecting the yield gradient in spite of the larger differences between tree yields.

## 1. Introduction

Olives are the main woody crop in Spain. Olive orchards cover 2.58 Mha, of which 96% is dedicated to oil olive production [1]. Most of the olive orchard area (∼76%) is currently planted according to the traditional model: two, three or four trunks per tree and wide spacing between trees. However, 24% of the area presents a major challenge to mechanized operations due to steep slopes. Only 56% of the area is considered to be suitable for mechanization under traditional orchards [2]. Cropping olives for oil has traditionally been performed in the Mediterranean basin. However, in the last decade, this practice has spread to other countries, such as Chile, where the area for this crop increased from 5000 ha in 2003 to 18,000 ha in 2013 [3].

Since the introduction of the trunk shaker, no new harvesting systems have been developed for olives [4]. Thus far, canopy shaker systems have been tested in traditional olive oil orchards in Spain. This harvesting method is characterized by a high amplitude and low frequency applied directly to fruit-bearing branches [5]. However, mechanical harvesting is still in the developmental stage. Currently, it is possible to observe more than 50 units of large continuous straddle harvesters operating in modern groves throughout the world: high and super high density groves with more than 1000 trees per hectare and one trunk per tree (Spain, Argentina, Chile, USA, and Australia) [6]. This solution requires a strong orchard and tree adaptation to the machine [7].

According to the MAX program (Conservation Technology Information Center, West Lafayette, IN, USA), machinery operation can be as high as 25% of the total cost of crop production. Agricultural machinery is seldom engaged in productive work 100% of the field time. Many delays occur that result in lost time, and any operation will vary greatly from field to field and farm to farm [8,9]. Effective field capacity and field efficiency are two primary parameters that are used to evaluate machinery performance. While the effective field capacity represents the amount of processing that a machine can accomplish per hour [10], the field efficiency is defined as the ratio between effective and theoretical machine capacities and relates the estimated and actual time that is required to complete a field operation (with no reference to the area) [11]. In the past, collecting and managing field data have been a significant component of human labor that is time consuming and labor intensive. Modern telecommunication technologies are required to improve the data collection efficiency and precision agriculture [12].

A large body of research has reported the use of a global system for mobile communication (GSM) and short message services (SMS) to conduct field operation data acquisition and has investigated the feasibility of this system [12–14]. The advantages of agricultural field operation data transmission through GSM system are (1) simple power solution; (2) coverage of a wide range of areas [15]; (3) maintenance of user data in the GSM service center for 24 h if the host server is out of service, and (4) group broadcast easily enabled sending real-time alerts from any dysfunctional devices for immediate attention. These technologies have been developed for tracking equipment transport vehicles, ambulances, fire, *etc.* with various data uses that are very different from those required in agriculture. In other cases, the devices require interventions that are far too costly for a fleet that is composed of multiple units [16].

Optimum machinery management is considered to be one of the main factors in making olive orchards more profitable and environmentally sustainable. In mechanized operations, at least two factors play a very important part in the effective field capacity. One factor is machine management, which involves such items as machine speed selection, labor force used, machine hours, machine geographical location, flow of material to and away from the machine, and maintenance information. The second factor involves the physical condition of the field, which includes field size and shape, topography, row length and orchard layout, row-end turning space, and surface condition in the turn area [17]. Overall, precision agriculture, particularly precise vehicle tracking systems, is considered to be essential to reach mechanized operation efficiency. These systems can avoid travelling over long distances to be on-site during operations in olive tree fields; therefore, Global Navigation Satellite Systems (GNSS) can reduce the time and cost for studies [18].

In agriculture, the use of a positioning satellite system to determine the real-time machine position is a reality. The new position location receivers that are available for agricultural operation combine multiple GNSS systems (at least with GPS & GLONASS) to provide better accuracy under canopy coverage. This improvement prevents problems due to GNSS outages under tree canopy and increases the overall performance improvement and robustness of satellite-based navigation, thus making it possible to obtain a better position fix within an orchard with large trees [19].

Furthermore, yield variability in herbaceous crops may arise due to soil characteristics. In olive groves, however, there is great variability among individual trees each year, although mainly in non-irrigated plants [20]. In addition, the occurrence of alternate bearing in olive trees makes it more difficult to interpret yield maps for fruit trees. Few studies have been conducted on yield mapping in woody crops. For hand-harvested citrus, yield maps [21,22] or canopy size maps [23] have been reported. Maja and Ehsani [24] developed a load-cell-based yield monitoring system for the Oxbo citrus mechanical harvesting machines, achieving a correlation of 0.97 between the actual weight and the computed weight with an average error of 7.81%.

According to Grisso *et al.* [25], both farmers and researchers can benefit from advances in real-time data geo-referenced data logging, which often can be reviewed off-site to examine traffic patterns, field practices, and other operational issues. Currently, the monitoring of agricultural field operation is now feasible and may be a useful tool for olive farmers, but as of yet, it is not widely used in commercial olive groves. Bakhtiari *et al.* [26] reported a savings range from 18% to 40% of the total non-working travelling distance for a combine harvester, making optimal mechanized operation planning.

Previous research on mechanized operation performance and field layout has shown a significant trend between forage harvester effective field capacity and crop yield, lengthwise slope and field area [27]. Some authors suggest that field shape influence on effective field capacity [28], and some studies have even determined that the optimal field shape should be rectangular with 4:1 length:width ratio [29].

The objective of this research was to determine the olive harvester field performance (effective field capacity and field efficiency) using a new remote and autonomous device in three olive harvesters and to evaluate a yield-monitoring system for a mechanical olive harvester that was fabricated for this study.

## 2. Materials and Methods

**Figure 1.**
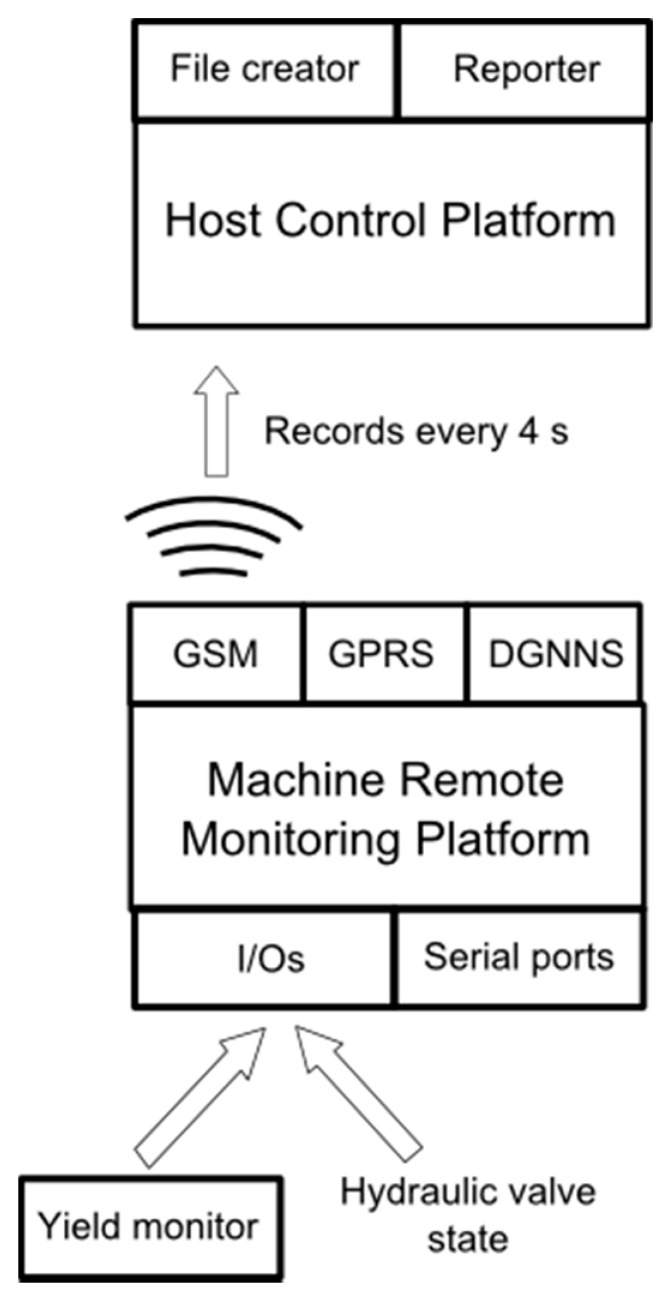
Hardware scheme of a remote wireless automatic monitoring system. Data transmission and communication between agricultural vehicles and expertise center.

All of the design decisions with respect to the developed telemetry tracking system were made with three criteria in mind: low-cost and study and scalable capabilities. The structure of the system that is presented in this research (Figure 1) can be divided into two major sections: the Machine Remote Monitoring Platform (MRMP) and the Host Control Platform (HCP) for monitoring, statistical analysis and field information reporting for decision-making.

### 2.1. Machine Remote Monitoring Platform (MRMP)

The MRMPs were located aboard on each olive harvester that was used in this study, and each MRMP was equipped with a terminal MTX 65+G (Matrix Electronica, S.L., Madrid, Spain) that was programmed using JAVA language. The terminal MTX 65+G integrated a GSM (Global System for Mobile communications) GPRS radio system and a DGNSS (Differential Global Navigation Satellite System) receiver with 16 channels, including a range of I/Os and USB/SPI/I^2^C/RS232 ports, was used to track the olive harvesters. The terminal had storage capacity to keep the data when GSM coverage was not available. In addition, it had a preprocess functionality to provide understandable packets to the HCP. The GSM module enables to the MRMP to transmit data packets in real time every 4 s, which will allow further analyses. Each data packet that was used in this study contained the agricultural vehicle identification machine (IM), date, time, latitude and longitude, altitude, speed, heading, coverage, and four digital and two analogical inputs with 12 bit. One digital input signal enabled the monitoring the status of the hydraulic valve to determine when the shaking system of the harvesters was working. One analogical input signal was used for the MRMP mounted on lateral canopy shaker to sense the accumulated fruit weight and to determine the orchard olive yield.

### 2.2. Host Control Platform (HCP)

To exploit the data that were generated by the system, two programs were used. These programs accomplish two functions: data storage and data consultation and downloading. These programs were programmed on Visual Basic and implemented in Excel (Microsoft Corp., Redmond, WA, USA). These data were used as input files directly downloaded, previously converting from coordinated universal time convention (UTC) to local time.

The first computer program was used to create a file Keyhole Markup Language (KML), which permits vehicle data rendering on Google Earth [30]. Each record is a “placemark”, which allows the examining of particular locations, and appearance characteristics may differ according the vehicle status sensors at the time. Clicking on the “placemarks” generates the associated information, that is, the content of the entire field that composes the record; latitude (y) and longitude (x) can always be read by another application window. In addition, these data indicate where the vehicle was at a particular time period and the status of the digital inputs. With these specific tools, it may be possible to determine the field works that are performed for the farm vehicle: field plots visited, time worked, surfaces worked and distances travelled. However, this determination would be time consuming and therefore costly whether it was performed manually by *in situ* technicians examining.

The second computer program that was developed was called “REPORTER”, which enables a rapid and easy analysis. Once the file to be processed is selected, the program create a results table in which each row refers to a worked plot, with the name in the first column, while the remaining columns are labeled with the different times used and distances traveled for the vehicle. To implement this program, it is necessary to previously compile a database of plots that are worked by the vehicle, with boundary map polygon information from ESRI’s shape files and a series of programming modules that are specialized in handling shp files, topology, projections of geographical coordinates, *etc.*

### 2.3. Yield Monitor

A prototype automatic system to record yield that was designed and fabricated specifically for this study was installed on a lateral canopy shaker (Oxbo 3210) that included a catch frame to perform an integral harvesting for traditional orchards. This system consisted of a controller box, and the force transducer (MLC807-3000kg, ManyYear Technology Co., Ltd., Hong Kong, China), which was installed in the rear receptacle support to measure its accumulated weight. This force transducer was wired to provide an analogical signal from 0 to 3.3 V and the circuitry was adjusted for an offset of zero volts. The procedure for calibrating the force transducer in the laboratory in accordance with the standards for linear measurements device (USBR 1045-89) was provided. The laboratory test procedures consisted of a first set of six loads from 40 to 240 kg (in 40-kg increments). These loads were sequentially loaded into the harvester receptacle and then unloaded. In a second set, a large known load weight of 288 kg was located into the receptacle. Incremental loads of 40 kg were then added to a total of 528 kg, after which the receptacle was unloaded. Load weights of 40 kg were used with the purpose of simulating the average kilograms per tree harvested under our expected conditions. Two repetitions were used for developing the calibration equation that will determine the estimated olive fruit load in the field tests.

In the field tests, the yield monitor system provided the accumulated weight of the harvested fruit in real-time and when the receptacle was downloaded. The weight data were processed after the harvester operation to obtain each tree harvest and were assigned to each harvested tree depending on the provided harvester location for each record. Fruit management delay along the catch frame belts was used to determine how long it takes the fruit to be stored in the receptacle. The data from the load cells together with the DGNSS data for the fruit receptacle locations were transmitted for the terminal unit wirelessly to the host control platform. The setup instructions and data were transmitted. The sequence of data transmission was DGNSS, weight, A/D, and RS232.

The GNSS sensor that was used for this work was integrated in the MTX 65+G Siemens terminal with output data in the NMEA-0183 GGA string via an RS-232-compatible serial port at 9600 bps.

Spatial distribution maps were created using the GIS software SStoolbox (SST Development Group, Inc., Stillwater, OK, USA) by interpolating the harvest of 33 trees using the inverse distance weighted method.

### 2.4. Time Study Methodology Validation

In the past, machinery field operation research was tedious and time consuming, requiring the travel of large distances and the researcher to be on-site during the operation. In this study, an automatic methodology was used to examine the time elements and to classify them into each field task. This methodology did not consider the harvester actions but instead classified time intervals depending on work parameters, such as speed, covered distance or status of the digital inputs (in this case, the hydraulic valve state). The automatic methodology was programmed on a computer using conditions on time elements to determine in which category operation the record would be included. The proposed methodology arranged the time elements into four categories:

1. Movement time: Time in which the machine was moving
  - Working time: Time in which the machine was performing the work it was designed to carry out.
  - Transport time: Time in which the machine was moving without performing the work it was designed to carry out.
2. Stoppage time: Time in which the machine was stopped but its engine was on.
3. Parking time: Time in which the machine was stopped and its engine was off.
4. Uncertainly time: Time during which the data that were provided by MRMP were inadequate or insufficient to discern what the machine was doing.

To evaluate the automatic methodology, a manual time division and data analysis was provided and compared to an automatic time elements classification. Automatic methodology employed a hydraulic valve state to separate working from transport time; speed was used to discern stoppage and parking time from movement time, and stoppage time was separated from parking time considering that the MRMP did not emit data when the machine engine was off.

The effective field capacity and field efficiency [31] were calculated using the automatic and manual methodology to test the appropriateness of the automatic methodology for tracking agricultural machinery. The travel times between fields and intervals with insufficient information regarding the harvester were omitted in the calculation process. The potential work parameters were obtained for each type of harvester to determine its appropriateness for each field operation. The displacement and preparation time elements of the machine were removed from the process.

(1) $$\text{Effective field capacity}\;(ha\;h^{-1})=\frac{\text{Harvested area}}{\text{In field total working time}}$$

(2) $$\text{Field efficiency}=\frac{\text{Effective field capacity}}{\text{Theoretical field capacity}}$$

### 2.5. Experimental Field and Harvesters

**Figure 2.**
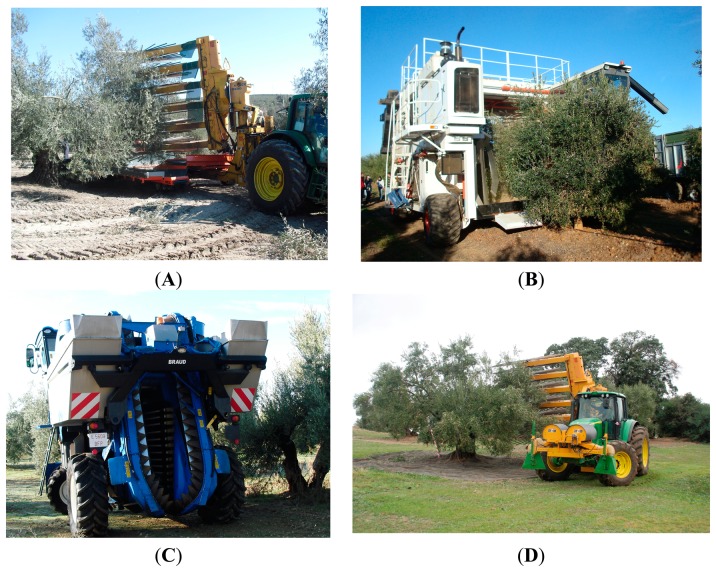
Tracked harvesters: (**A**) Oxbo 3210 with catch frame; (**B**) Maqtec, Colossus; (**C**) New Holland VX 7090 and (**D**) Oxbo 3210 without catch frame.

Field tests were conducted in commercial olive orchards in southern Spain (Latitude: 37.9192028 N, Longitude: 4.7207889 W) and Chile (Latitude: 35.2967166 S, Longitude: 71.4126333 W) to evaluate ability to record remote information from harvesters yielding the ability to characterize the farming operation. Three commercial olive harvesters were tracked during the 2010–2011 and 2011–2012 olive harvesting seasons in southern Spain and northern Chile. The harvester models were the following: MaqTec, Colossus (MaqTec, Venado Tuerto, Santa Fe, Argentina) integral self-propelled harvester for high density olive orchards; Oxbo, 3210 (Oxbo International Corp., Kingsburg, CA, USA) non-integral tractor drawn harvester for high density orange orchards; and New Holland, VX7090 (CNH Global, Burr Ridge, IL, USA) integral self-propelled harvester designed for vineyard and super high density orchards (Figure 2). A New Holland VX7090 harvester was used on a super-high-density olive orchard (more than 1000 trees/ha hedgerow trained). The self-propelled MaqTec, Colossus straddle harvester was used on a high-density olive orchard (285–830 trees/ha single trunk trained), located in Córdoba, Spain. The tractor drawn Oxbo, 3210 harvester was used in two configurations: (i) in a high-density olive orchard (400 trees·ha^−1^ hedgerow trained) without a catch frame and (ii) in a traditional olive orchard (70 trees· ha^−1^, quincunx spacing, several trunks trained) with a catch frame that was designed at the University of Córdoba (Table 1).

### 2.6. Field Representation and Data Analysis

A field area is represented as a closed loop, 2D polygon and is stored in shape-files with associated informational attributes that describe the geometrical field representation. The field characteristics, such as feature geometry (regular, typical and irregular), angle between headland and row (perpendicular, perpendicular-acute and acute), row length and alley width, were measured for both the straddle harvester and hedge straddle harvester in the 2011/2012 harvesting season. Regular geometry indicates an approximately rectangular or squared field, which has some irregularities, and irregular geometry indicates a triangular or irregular shaped field. All of the characteristics were compared based on field efficiency except for the row width, which depended on the effective field capacity. A homogeneous work unit was defined for each case as one workday or a day fraction when the considered field characteristic value did not change significantly.

SPSS (IBM, Armonk, NY, USA) and Statistix 8 (Analytical Software, Tallahassee, FL, USA) were used for the statistical analyses.

## 3. Results and Discussion

### 3.1. Time Elements Methodology Validation

**Figure 3.**
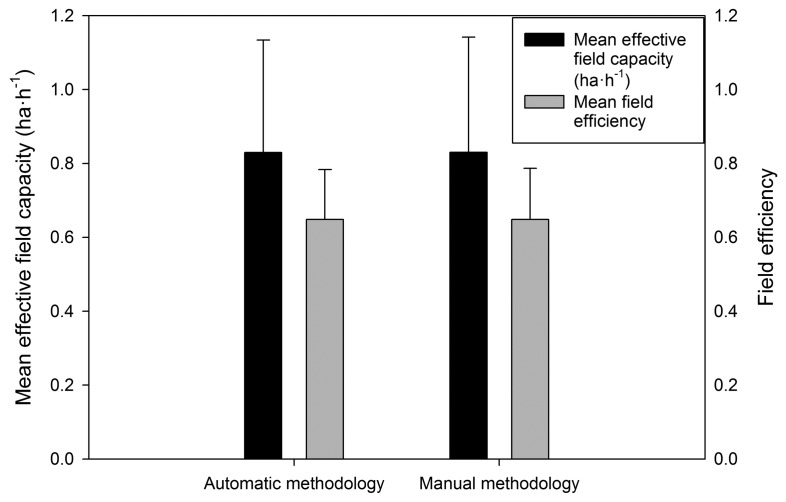
Effective field capacity and field efficiency calculated using automatic and manual methodologies. Different letters show significant differences between groups according to Student’s *t*-test (*p* < 0.05).

Of the 781 h that were recorded for the hedge straddle harvester, 720 h that corresponded to the season 2011–2012 with a high temporal resolution (4 s) were used to validate the new methodology. This methodology was compared to the manual calculation of the time element. Figure 3 shows the mean and standard deviation values that were obtained for the new and manual methodology for effective field capacity and field efficiency.

The aim of this study was to validate this new methodology in an experimental setting. No significant differences were found between the methodologies. Therefore, these preliminary results indicate that the methodology may be appropriate for use in agriculture machinery tracking while improving the work efficiency of technicians by reducing their time in the field.

### 3.2. Effective Field Capacity and Field Efficiency

In this study, the recorded tracking time was 11 h for the lateral canopy shaker, 257 h for the straddle harvester and 781 h for the hedge straddle harvester. The hedge straddle harvester stands out for its highly effective field capacity, with one season in Spain with 0.70 ha·h^−1^ (SD ± 0.1) and two seasons in Chile with 0.74 ha·h^−1^ (SD ± 0.2) and 0.83 ha·h^−1^ (SD ± 0.3). However, the highest field efficiency was achieved by the lateral canopy shaker (Table 2). This machine was a non-integral harvester and did not suffer from time losses when unloading fruit. Furthermore, this machine is smaller in size and weight. The straddle harvester obtained low values of the effective field capacity and field efficiency, most likely due to the dampness of the 2010–2011 harvesting season in the south of Spain (from October 2010 to March 2011, the average relative humidity was 71.7%, and the total rain was 865 mm). The straddle harvester was the most voluminous (4.0/6.83 × 8.08 × 4.35/4.68, width × length × height) and heaviest (28 tons) indicate that the working and travelling speeds were very slow. The orchard topography, which was not completely flat, may also have influenced the low values that were shown by the straddle harvester. In Australia, this harvester had an effective field capacity of 0.30 ha·h^−1^ [32]. Nevertheless, an effective field capacity of 0.30 ha·h^−1^ is lower than that of the other harvesters operating under our conditions. The low speed directly affects the effective field capacity; however, in an integral harvester, this affect means more time spent shaking the olive tree, which could lead to the additional felling of fruit (out of the scope of this work).

Tracked canopy shakers improve the effective capacity of conventional harvesting methods, which usually varies from 0.12–0.20 ha·h^−1^ [33], as reported for tractor-hitched trunk shakers, or from 0.25 to 0.30 ha·h^−1^, as measured for self-propelled trunk shakers [34]. In Australia, a COE L2-E Receiver (3453, Riviera Rd., Live Oak, CA, USA) side-by-side harvester showed field capacities of approximately 0.39 ha·h^−1^ [32], and in Italy, canopy shakers with a catch frame for high-density olive orchards can harvest 0.25 ha·h^−1^ [35]. In previous tests, a lateral canopy shaker without a catch frame was used on traditional olive orchards; working around tree canopies, this shaker harvested 0.39 ha·h^−1^. This machine can also make-crossed rounds to harvest a square spaced olive orchard, and its effective field capacity is 0.23 ha·h^−1^ [36]. Traditional olive orchard competitiveness could be improved using a canopy shaker to perform integral harvesting to similar levels of intensive olive orchards.

### 3.3. Field Characteristics Influence the Harvesting Operation

The integral harvester field efficiency was influenced by both the row length and down-the-row speed. These factors reduce the turning time and unloading elements when the harvester used the receptacle to store harvested fruit. When this storage occurs, the row length is limited by the receptacle storage capacity as related to the row production per length unit. At this point, the optimal orchard design may permit the harvesting of two rows before unloading to perform this operation only at one end of the row.

**Figure 4.**
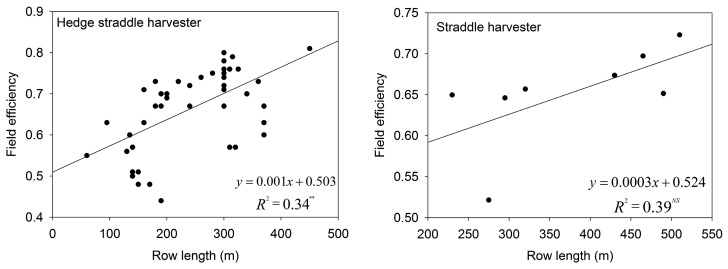
Trend between the effective field capacity and row length for both the hedge straddle harvester (**Left**) and the straddle harvester (**Right**).

The row length was significantly related to the field efficiency for the hedge straddle harvester but not to the straddle harvester for high-density olive orchards. This result was due to the straddle harvester performing less homogeneous work units than the hedge straddle harvester. The data scatter was very similar in both cases (Figure 4). Our results agree with the results that were reported by other authors regarding the influence of geometry on the effective field capacity [27,37].

The data in Table 3 show the mean and standard deviation for field efficiency according to the field shape. For the hedge straddle harvester, the geometry has significantly influenced the field efficiency, while for the straddle harvester, significant differences were not found. Field size and geometry also affect labor organization because if the row length varies, the driver must change the number of harvested rows to unload the receptacles, or the driving pattern must be changed.

Table 3 shows no significant differences between the regular and standard geometry for both of the harvesters. These data show that the angle between the headland and perpendicular row increased the field efficiency, thereby influencing the turning time elements and work organization when the angle was acute; more frequently, the workers used loop driving patterns to increase the turning radius. The hedge straddle harvester had significant differences between orchards that had a perpendicular angle between the headland and row at both ends and the others orchards that had an acute angle between the headland and row at both ends. These results agree with those of Shamshiri *et al.* [38], who noted that the turning time was greatly influenced by the field size, shape and driving pattern. Irregular field shapes with rows not intersecting the field boundary at a right angle presented additional turning problems.

The effective field capacity and field efficiency were affected by the row alley width. The hedge straddle harvester worked at different row alley widths, while the straddle harvester for high densities worked at similar alley widths. Therefore, the hedge straddle harvester was the only machine that provided data to analyze the alley width influence on the effective field capacity.

**Figure 5.**
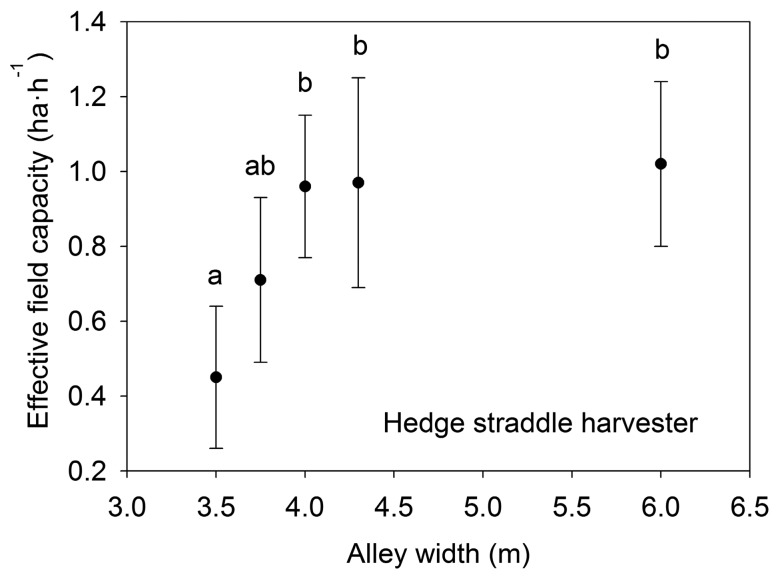
Effective field capacity and row alley width relationship. Different letters indicate significant differences (*p* < 0.05) according to Scheffé’s test.

The hedge straddle harvester provided significant differences between 3.5 m and 4 m or greater row alley widths. When the row alley was wide enough, this harvester easily made machinery paths, reducing turning time elements. With a high row alley width, the harvester would provide a high effective field capacity. Nevertheless, these differences were not significant when the row alley width was greater than 4 m, most likely because in super-high-density olive orchards, the vegetative row width and production increased when row alley width increased, thus reducing the harvester work speed and affecting the effective field capacity (Figure 5).

### 3.4. Yield Mapping

**Figure 6.**
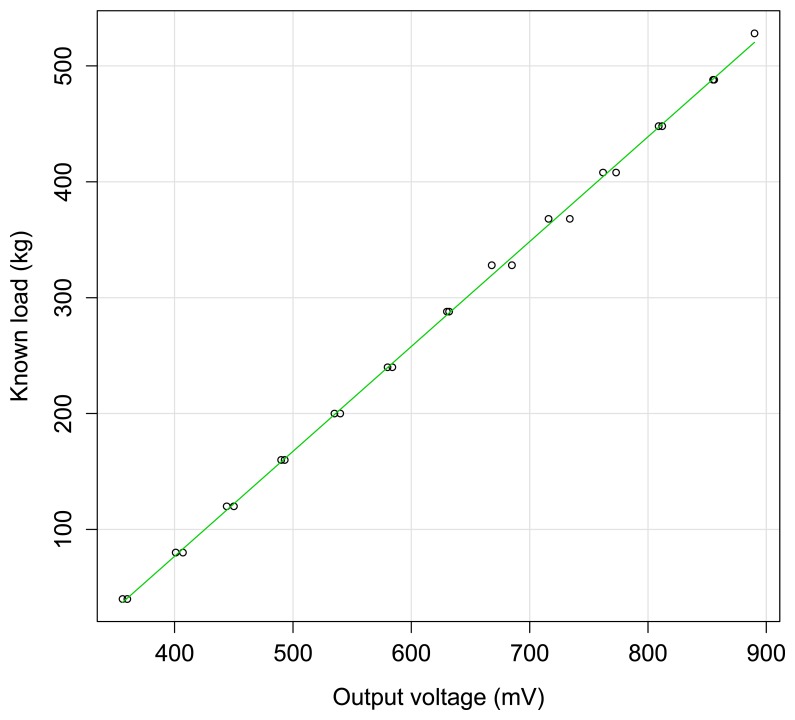
Relationship between the output voltage in the load cell and the known load weight.

The yield monitoring system performed very well in laboratory tests. Figure 6 shows a linear relationship between the output voltage and the known loads (*R^2^* = 0.9991, *p* < 2.210^−16^). The straight-line least-trimmed squares exhibited the following relationship:

(3) y=0.9046x−284.94

where *y* is the known loads (kg) and *x* is the output voltage of the load cell (mV).

This equation was used to obtain the estimated olive fruit weight per tree in the field tests. The x-intercept is (315.0) due to the support of the harvester receptacle on the load cell. The value 315 mV is the load cell output for the empty receptacle, and the offset adjustment load cell output voltage corresponded to 284.94 kg. Due to the constraint in the harvester receptacle as limited by the transport capacity, the maximum weight that can be loaded per test run cannot exceed 600 kg.

In the field for preliminary results, thirty-three olive trees were harvested from the orchard. The yield monitor provided a realistic estimate of the yield differences between olive trees, with average values of 41.24 kg per tree and standard deviation of 20.13 kg per tree. This high standard deviation was due to the irregularity of the trees in the traditional orchards. In the same area of study, variations over 50% in fruit load per tree were found in an olive orchard [39]. The yield assignment to each tree and the structural variations in vegetation are crucial pieces of information for constructing prescription maps for olive orchards. Using these precision farming techniques assists decision-making systems, allowing for variable-rate input application.

**Figure 7.**
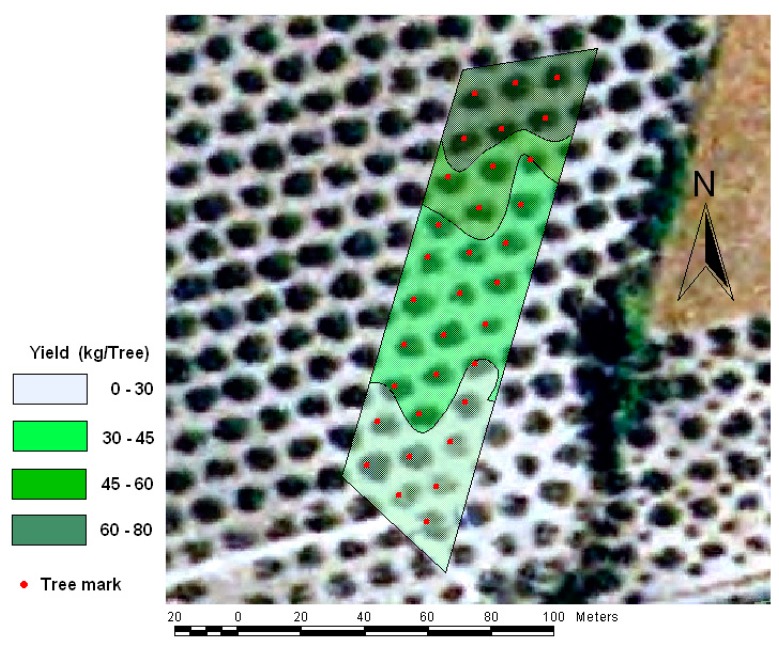
Olive fruit yield map estimate in a conventional orchard system.

A potential application of a telemetry system in combination with yield monitoring is olive fruit yield mapping in real time as shown in Figure 7. The performance of the olive fruit yield monitor ranged between 8.4 kg per tree and 85.83 kg per tree. High variability orchard plot was chosen to test yield monitor in order to demonstrate its performance in highly changeable conditions. This map presents a gradient of decreasing production from northwest to southeast. The altitude decreased from the northwest corner to the southeast corner, which was the lower point in the map on the south stream end. Based on the author’s assumptions and farm technician consultation, this decrease was due to fungal disease attacks, mainly by olive tree peacock leaf spot (*Fusicladium oleagineum*) and anthracnose infections (*Colletotrichum gloeosporioides*). The map information could be used to develop models that describe the relationship between disease severities and yield (kg/tree). In addition, these models could be used to optimize the control of fungal diseases.

A small-scale yield contour map was generated using a kriging for a conventional orchard system. This study demonstrates the possibility of identifying localized zones for site-specific application. Results such as these indicate that such technologies could be implemented on real harvesters and, in the near future, could be used on commercial farms. However, further studies are required to determine whether these techniques would be profitable for use in olive groves, even on small farms, where economic efficiency must be achieved [20].

## 4. Conclusions

An agriculture vehicle-mounted telemetry system, which automatically determines the labor time, allows a large amount of data to be processed, and an olive fruit yield monitoring system was successfully developed and operated. Both systems were successfully implemented on an olive fruit harvester and operated in commercial olive orchards.

The following conclusions were drawn based on the results of this research:

- An optimized process was validated to determine the time element and to obtain satisfactory machine capacity and efficiency. The use of this automatic process and the developed prototype telemetry system promotes the following: (i) reduced travel time because technicians can oversee the machinery from the management center; (ii) improvements in the immediate accessibility of machinery information because data are available on a web server and (iii) cost savings because these systems improve the work efficiency of technicians, and these systems can be used to optimize the in-field machinery management. Additionally, work organization can be improved using precision agricultural techniques, such as agricultural fleet management, to achieve acceptable farm organization in economic and environmental terms.
- The super-high-density straddle harvester achieved higher machine capacities (0.7–0.8 ha·h^−1^) than did the other harvesting methods, but this machine only works in super-high-density olive groves. Using a lateral canopy shaker, traditional olive orchard harvesting performance achieved higher effective machine capacity values (0.88) than did the other harvester.
- The yield monitoring system, which uses load cells that are mounted below the harvester receptacle, was developed and tested under laboratory and simple field conditions. The system performed well in the laboratory test with a linear relationship between the output voltage and the known loads (*R*^2^ = 0.9991, *p* < 2.210^−16^). Although each tree yield could be discerned in the presence of great variability and the results of the field test also provided a good accuracy, further studies are needed to obtain reliable results and to enable commercial yield mapping for olive groves.

One of the great advantages of this system is the ability to connect field system with expertise centers located at distant geographical sites. The results of this study indicate that such technologies could be used on commercial farms. However, for better implementation, further studies are required to determine whether these techniques would be profitable for use in olive orchards, even on small farms, where economic efficiency must be achieved.

## Figures and Tables

**Table 1.** Detailed description of the tracked harvesters, and commercial field harvested.

**Harvester**	**Harvester Typology**	**Olive Orchard Typology**	**Location**	**Harvesting Season**
MaqTec, Colossus	Straddle harvester	High density (285–830 trees·ha^−1^)	Spain	2010–2011
New Holland, VX7090	Hedge straddle harvester	Super high density (>1000 trees·ha^−1^)	Spain and Chile	2010–2011 and 2011–2012
Oxbo, 3210	Lateral canopy shaker	High density (400 trees·ha^−1^)	Spain	2010–2011
Oxbo, 3210 + catch frame	Lateral canopy shaker	Traditional (70 trees·ha^−1^)	Spain	2011–2012

**Table 2.** Effective field capacity and efficiency for tracked olive harvesters. Values are mean ± standard deviation (maximum/minimum range).

**Harvester**	**Harvesting Season**	**Tracking Time (h)**	**Effective Field Capacity (ha·h^−1^)**	**Field Efficiency**
Lateral canopy shaker with catch frame	2011–2012	1.5	0.36	0.71
Lateral canopy shaker	2010–2011	11	0.36 ± 0.12 (0.5/0.3)	0.88 ± 0.12 (0.9/0.7)
Straddle harvester	2010–2011	257	0.15 ± 0.05 (0.2/0.1)	0.63 ± 0.13 (0.8/0.5)
Hedge straddle harvester in Spain	2010–2011	38	0.70 ± 0.1 (0.8/0.6)	0.60 ± 0.07 (0.7/0.5)
Hedge straddle harvester in Chile	2010–2011	23	0.74 ± 0.2 (0.9/0.5)	0.75 ± 0.12 (0.9/0.6)
Hedge straddle harvester in Chile	2011–2012	720	0.83 ± 0.3 (1.3/0.2)	0.65 ± 0.13 (0.9/0.2)

**Table 3.** Field efficiency based on the field shape and the angle between the headland and row.

**Hedge Straddle Harvester**		**Field Efficiency**	

**Factor**	**Category**	**Mean** *	**Std. Dev.**
Field geometry	Regular	0.69 ^b^	0.11
	Standard	0.61 ^b^	0.15
	Irregular	0.56 ^a^	0.15

Angle between the headland and row	Perpendicular/both ends	0.69 ^b^	0.11
	Perpendicular/acute	0.61 ^b^	0.10
	Acute/both ends	0.43 ^a^	0.20

**Straddle Harvester**		**Field Efficiency**	

**Factor**	**Category**	**Mean** *	**Std. Dev.**

Field shape	Regular	0.66 ^a^	0.08
	Standard	0.60 ^a^	0.10

* Mean values with the same grouping letter are not significantly different (*p* < 0.05) by the non-parametric Wilcoxon test;

a Significant higher values;

b Significant lower values.
